# Pilot Study of Community Group Mindfulness Training

**DOI:** 10.1192/bjo.2024.465

**Published:** 2024-08-01

**Authors:** Alexandra Cornwall, Naz Sergison, Peter L Cornwall

**Affiliations:** 1 Bestill for mind and body, Redcar, United Kingdom; 2 Tees, Esk and Wear Valleys NHS Trust, Middlesbrough, United Kingdom

## Abstract

**Aims:**

Community mental health transformation relies on the integration of NHS, local authority, and voluntary agencies to deliver mental health care and support where and when people need it. There is a concern that resources may be diverted to services focused on those with less severe problems and without robust outcome data. We plan to develop a network of self-sustaining mindfulness support groups in a disadvantaged locality with very limited community resources. We provided a pilot mindfulness programme to a group of mothers of primary school age children in East Cleveland.

**Methods:**

Participants were recruited through poster adverts at a primary school. The programme was delivered through 12 weekly hour-long sessions at the school. The group facilitators had basic training in mindfulness. The aim was to teach basic mindfulness practices that could be used in everyday life, including breath work, meditation, and journalling. Mental health status at baseline, mid-point (week 6) and end point (week 12) was measured using the GHQ-12 (score 0–36 and a threshold for likely psychiatric disorder). Data was analysed using *t* test for continuous scores and χ^2^ test for caseness.

**Results:**

14 women responded to the invitation and 9 completed the programme attending a mean of 11.2 sessions. The mean age of participants was 37.4 years and 8 reported previous mental health treatment with medication or psychological therapy, with 4 currently taking medication, but none were known to secondary mental health care services. GHQ-12 scores at baseline indicated significant levels of mental health distress (mean score = 24.1, caseness = 100%). At the midpoint there was a 56.2% reduction in GHQ-12 scores, and this increased to 62.0% at the endpoint. 2 participants remained GHQ cases at both follow-up assessments. The improvement was highly significant (baseline mean score (SD) = 24.1 (2.71); final mean score (SD) = 9.11 (6.15); paired *t* test: *t* = 7.23, df = 8, *P* = 0.0001).

**Conclusion:**

This was a novel programme where participants gained access through being parents of primary school aged children. Despite being an unselected community sample, the participants reported significant levels of psychological distress. This highlights both that most people with mental health problems have no contact with psychiatric services and that there remains a high level of unmet need in the community. In this sample, a remarkable level of improvement was demonstrated from a relatively simple and straightforward intervention. Clearly, this will benefit from replication in greater numbers in more diverse samples and settings and with follow-up to see if the benefits persist beyond the intervention phase.

